# Roles of MMP-2 and MMP-9 and their associated molecules in the pathogenesis of keloids: a comprehensive review

**DOI:** 10.3389/fphar.2024.1444653

**Published:** 2024-11-25

**Authors:** Yajie Wang, Liying Zheng, Lai Zhang, Yuncheng Tai, Xuesong Lin, Zhencheng Cai

**Affiliations:** ^1^ Department of Burn Surgery, Taizhou Central Hospital (Taizhou University Hospital), Taizhou, Zhejiang, China; ^2^ Postgraduate Department, First Affiliated Hospital of Gannan Medical College, Ganzhou, China; ^3^ Department of Orthopedics, Taizhou Municipal Hospital, Taizhou, Zhejiang, China

**Keywords:** keloids, gelatinases, MMP-2, MMP-9, molecular mechanism

## Abstract

Keloid scars (keloids), a prototypical form of aberrant scar tissue formation, continue to pose a significant therapeutic challenge within dermatology and plastic surgery due to suboptimal treatment outcomes. Gelatinases are a subgroup of matrix metalloproteinases (MMPs), a family of enzymes that play an important role in the degradation and remodeling of the ECM (a pivotal factor for keloids development). Gelatinases include gelatinase A (MMP-2) and gelatinase B (MMP-9). Since accumulating evidence has shown that gelatinases played a crucial role in the process of keloid formation, we summarized the current knowledge on the association between MMP-2 and MMP-9 expression and the pathological process of keloids through a comprehensive review. This review demonstrated that the interplay between MMP-2, MMP-9, and their regulators, such as TGF-β1/Smad, PI3K/AKT, and LncRNA-ZNF252P-AS1/miR-15b-5p/BTF3 signaling cascades, involved in the intricate balance governing ECM homeostasis, collectively driving the excessive collagen deposition and altered tissue architecture observed in keloids. In summary, this review consolidates the current understanding of MMP-2 and MMP-9 in keloid pathogenesis, shedding light on their intricate involvement in the dysregulated keloids processes. The potential for targeted therapeutic interventions presents promising opportunities for advancing keloid management strategies.

## 1 Introduction

Keloid scars (keloids), a type of abnormal scar tissue formation, remain a therapeutic challenge in the field of dermatology and plastic surgery. The characteristic of keloids is the dysregulated fibroproliferation, excessive production of extracellular matrix (ECM), and extension beyond the initial wound (Khattab et al., 2022). Patients suffering from keloids often feel pruritus and pain, which cause immense physical and mental problems and profoundly impair the quality of life (Tian et al., 2023). Epidemiological studies have demonstrated a higher prevalence of keloids among females. Furthermore, individuals of African and Asian descent, particularly those with darker skin complexions, exhibit a greater incidence of keloid formation. The estimated prevalence of excessive scarring was 2.4%, 1.1% and 0.4% in Black, Asians and Caucasians, respectively (Lee et al., 2023). Therefore, an in-depth study on keloids is of profound significance. Currently, the pathogenesis of keloids remains unclear. Although there are various clinical treatment methods available, none of them can fundamentally cure keloids. In addition, keloids are highly susceptible to recurrence, and the keloids continue to grow and invade surrounding normal tissues.

The pathogenesis of keloids is complex, which is a confluence of multiple contributing factors. A lack of animal models has limited investigational studies into exact pathological mechanism of keloid formation (Ekstein et al., 2021). Scar formation and tissue regeneration are essential processes of organism repair injury (Wei et al., 2020). The wound healing processes leading to tissue repair and regeneration are generally divided into four phases: hemostasis, inflammation, proliferation, and remodeling (Sathiyaseelan et al., 2023). The recruitment of inflammatory cells and fibroblasts contribute to scar remodeling in the early phase of wound healing (Perez et al., 2017). Specifically, fibroblasts create a collagen-containing ECM that is balance of synthesis and degradation (Li et al., 2023; Van Haaften et al., 2020). Therefore, an imbalance between collagen production and ECM degradation contributes to scar formation. Many researchers have found that the excessive ECM production is closely associated with a decrease or increase of matrix metalloproteinases (MMPs), especially gelatinases (Panichakul et al., 2022; Seyed et al., 2023).

MMPs are a family of zinc-dependent endopeptidases, and they share a common structural motif, known as the catalytic domain (Anchan et al., 2022). This domain contains a catalytic zinc ion required for their enzymatic activity (Bonvicini et al., 2014). MMPs are divided into different subgroups based on their substrate specificity and domain structure. Some common subgroups include collagenases, gelatinases, stromelysins, and membrane-type MMPs (Li et al., 2022). MMPs play a crucial role in the degradation and remodeling of the ECM. The ECM is a complex network of proteins and carbohydrates that provides structural and biochemical support to cells (Caon et al., 2020). MMPs are responsible for breaking down various components of the ECM, allowing for tissue remodeling, wound healing, and other physiological processes (Kim et al., 2022; Zhang et al., 2023). Recently, MMPs have been shown to participated in the pathogenesis of many diseases, including keloids, idiopathic pulmonary fibrosis and various tumors (Chen et al., 2022; de Almeida et al., 2022; Herzog et al., 2019). Meanwhile, MMPs have been proposed as appropriate therapeutic targets for many diseases (Craig et al., 2015; Liu et al., 2023). Luo et al. (2023) reported that oleanolic acid significantly suppressed keloid fibroblast proliferation and reduced ECM deposition by increasing the level of MMP-1, suggesting that oleanolic acid might be a potent drug for treatment of keloids. Similarly, Jeon et al. (2016) also found that hepatocyte growth factor can be used to treat keloids by increasing MMP-1 expression. Further study showed that rats were treated with MMP-1 by intraperitoneal injection significantly reduced scar formation (Keskin et al., 2021). Some studies showed that gelatinases were also involved in the progression of keloids. In recent years, the role of gelatinases in keloids have received increasing attention of researchers. In this review, we mainly summarize the current knowledge about gelatinases in the progress of keloids.

## 2 The overview of gelatinases

Gelatinases are a subgroup of MMPs, a family of enzymes that play an important role in the degradation and remodeling of the ECM (Kalev-Altman et al., 2022). Gelatinases specifically degrade both gelatins and collagens, and they are involved in various physiological and pathological processes (Sukhonthasilakun et al., 2023). Gelatinases include gelatinase A (MMP-2) and gelatinase B (MMP-9). MMP-2 is a key enzyme involved in the degradation of gelatin, collagen, and other ECM components (Ekstein et al., 2021). MMP-2 is produced in a latent form and needs to be activated to perform its enzymatic function (Ekstein et al., 2021). MMP-9 is another enzyme that specifically targets gelatin and other ECM proteins (Hur et al., 2022). Like MMP-2, MMP-9 is secreted in an inactive form (Hur et al., 2022). The activation of MMP-2 and MMP-9 typically involves the removal of a propeptide domain (Jang et al., 2022). Several factors, including tissue inhibitors of metalloproteinases (TIMPs) and other proteases, are involved in this activation process (Eiro et al., 2023). TIMPs are a specific endogenous inhibitor and block access to ECM substrates by binding to the active site of MMPs (Robert et al., 2016). As a receptor of MMPs, TIMP-2 connects MMPs with membrane-type matrix metalloproteinase-1 (MT1-MMP) (Sato and Takino, 2010). TIMP-2 can promote the activation of MMPs proenzyme when MT1-MMP is removed from the binding of TIMP-2 (Sato and Takino, 2010). Gelatinases play essential roles in tissue remodeling, wound healing, and organ homeostasis by effecting angiogenesis, tissue repair, and cell migration (Aksoy et al., 2019; Ravanti and Kahari, 2000). Dysregulation of gelatinases is associated with various diseases, including keloids (Hao et al., 2018; Ulrich et al., 2010). For example, overactivity of gelatinases has been linked to excessive tissue degradation in diseases such as cancer metastasis, arthritis, and tissue fibrosis (Orsolic et al., 2020). On the other hand, insufficient gelatinase activity can lead to abnormal tissue repair and chronic inflammation (Brilha et al., 2018).

The regulation mechanism of gelatinases involves a complex interplay of various factors. The expression of gelatinases, like other MMPs, is modulated by transcriptional and posttranscriptional regulation. It was reported that inflammatory signals could increase MMP-2 and MMP-9 gene expression by activating transcription factors like AP-1, NF-κB, and SP-1 (Aljada et al., 2001; Shen et al., 2022; Yang et al., 2022). Fan et al. (2022) found that Kangfuxiaoyanshuan, a Traditional Chinese Medicine formulation, alleviated inflammation by inhibiting the NF-κB activation through decreasing phosphorylation of p65, resulting in reduced expression of TGF-β and MMP-2. In addition, growth factors and cytokines, such as TGF-β, EGF, and TNF-α, were also reported to modulate the expression and activity of gelatinases (Kondapaka et al., 1997; Rajashekhar et al., 2014; Tian et al., 2007). They can stimulate or inhibit MMP-2 and MMP-9 production through various signaling pathways, depending on the context and cell type (Kondapaka et al., 1997; Rajashekhar et al., 2014; Tian et al., 2007). For example, TNF-α has been proven to play an essential role in herpes simplex keratitis by stimulating MMP-2 and MMP-9 activities through the activation of FAK/ERK signaling in human corneal epithelial cells (Yang et al., 2012). It has also been shown that epigenetic modifications, such as DNA methylation and histone acetylation, affect gelatinase expression by influencing the accessibility of the MMPs gene promoter regions to transcription factors (Duraisamy et al., 2017; Liang et al., 2022). Studies showed that MMP-2 and MMP-9 can influence the overall MMPs activity by cleaving and activating latent forms of other MMPs, which indicated that gelatinases themselves, along with other MMPs, can participate in feedback loops (Kim et al., 2014).

**FIGURE 1:**
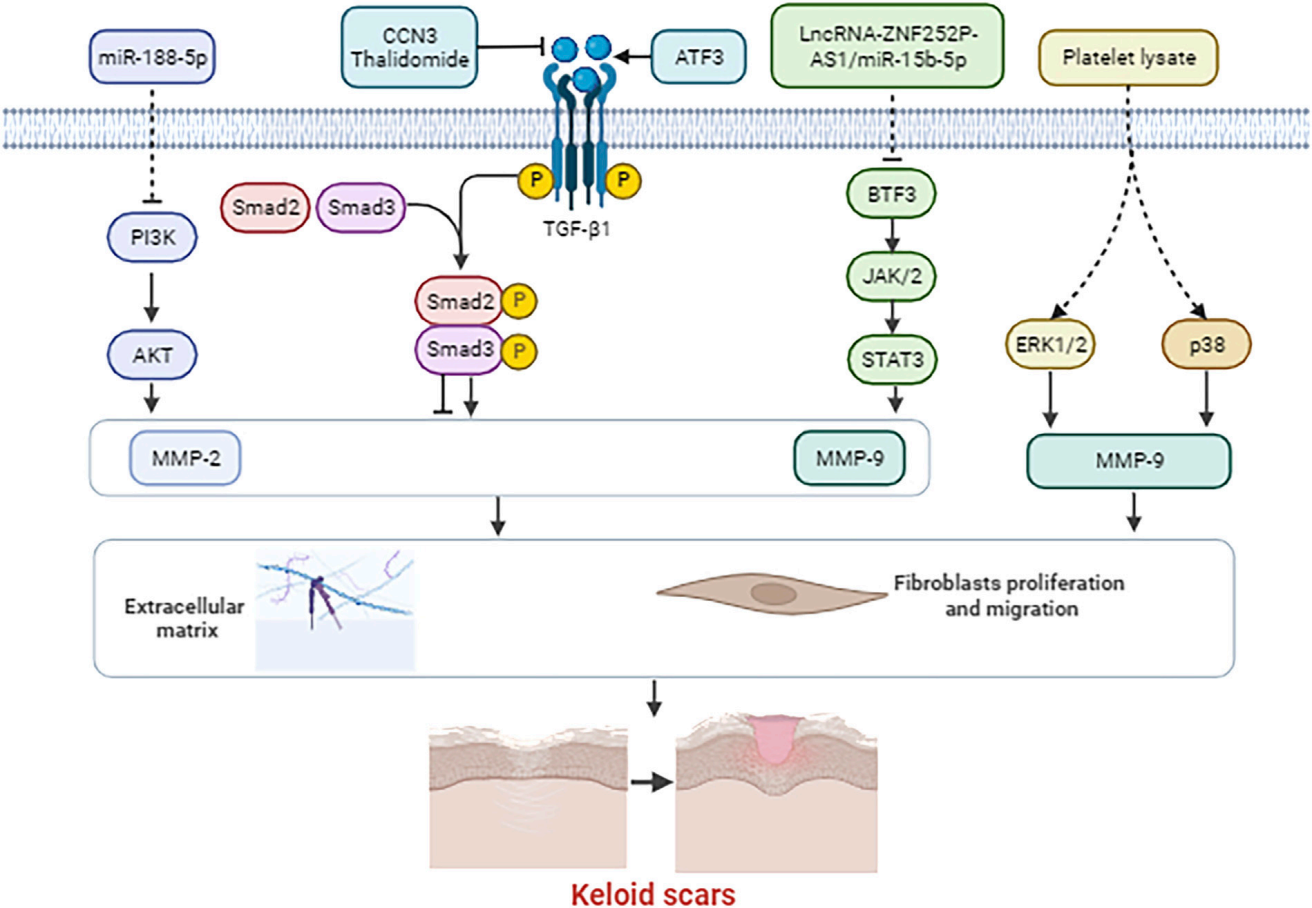
The interactions between MMP-2 and MMP-9 and their targeted gene/proteins and signal pathways.

Numerous studies have demonstrated that MMP-2 and MMP-9 are critical in cell proliferation, differentiation, apoptosis and angiogenesis, and are extensively implicated in the pathogenesis of various diseases, including neurological diseases, diverse tumors, and inflammatory conditions. Studies suggested that level of MMP-2 and MMP-9 significantly increases in the brain after stroke (Kumar and Patnaik, 2018). However, inhibition of MMP-2 and MMP-9 confers neuroprotection in stroke (Kumar and Patnaik, 2018). Lambert et al. (2003) reported that the expression of MMP-2 and MMP-9 were increased in human choroidal neovascularization occurring during the exudative most aggressive form of age-related macular degeneration. Additionally, inhibition of MMP-2 and MMP-9 could reduce angiogenesis (Lambert et al., 2003). Further, Hwang et al. (2021) demonstrated that salinomycin suppressed TGF-β1-induced EMT by inhibiting MMP-2 and MMP-9 via AMPK/SIRT pathway, thereby inhibiting the cell migration and invasion of lung cancer. Consequently, MMP-2 and MMP-9 may influence the progression of keloids through multiple signaling pathways, either promoting or inhibiting their development. Figure 1 shows the interactions between MMP-2 and MMP-9 and their targeted gene/proteins and signal pathways.

## 3 The roles of gelatinases in the progress of keloids

### 3.1 Regulation of extracellular matrix (ECM) and collagen

#### 3.1.1 Positive association between TGF-β1 and MMP-2/MMP-9

The ECM plays an important role in the development of keloids for its functions of structural support, cell adhesion, regulation of cell signaling, mechanical stress response, and impaired remodeling (Kim and Kim, 2024). Collagen plays multiple crucial roles in the ECM through tensile strength, shape and integrity maintenance, and regulation of cell adhesion and cellular signaling. Currently, mounting studies have identified the potential effects of MMP-2 and MMP-9 and their targeted proteins/signal pathways on the interactions of ECM or collagen molecules. TGF-β1 plays a significant role in the development and progression of keloids. TGF-β1 is a cytokine that is involved in various cellular processes, including tissue repair and fibrosis. TGF-β1 is a potent stimulator of fibroblast proliferation and collagen production. As we all known, the overactivation of fibroblasts can lead to excessive collagen deposition. TGF-β1 has been proved to a key factor in promoting this abnormal fibroblast activity. Additionally, TGF-β1 induces the synthesis of collagen, particularly type I and type III collagen, which are major components of the ECM. Studies showed that TGF-β1 is involved in the remodeling of the ECM by upregulating the expression of MMPs and TIMPs. Zhang et al. (2017) demonstrated that activation of the TGF-β1 by either mechanical stress significantly attenuated fibroblasts cell proliferation and ECM components by increasing the MMP2/TIMP2 mRNA ratio. Increasing evidence suggests that CCN3 is a negative regulator of the ECM (Ren et al., 2014; Yin et al., 2023). Liu et al. (2018) reported that TGF-β1 significantly decreased the expression of MMP-2 and MMP-9, and increased the expression of TIMP-1 in human mesangial cells. Furthermore, TGF-β1 significantly increased the accumulation of ECM (Liu et al., 2018). Importantly, overexpression of CCN3 attenuated TGF-β1-induced changes in MMP-2, MMP-9 and TIMP-1 (Liu et al., 2018). These results indicated that CCN3 inhibits accumulation of ECM by regulating the expression of MMP-2, MMP-9 and TIMP-1 via the regulation of TGF-β1. A recent study reported that TGF-β1 inhibitor significantly inhibited the development of cardiac fibrosis in mutant mice by blocking the expression of SMAD proteins, MMP-2 and MMP-9 (Subramanian et al., 2022). Sadick et al. (2008) demonstrated an increased expression of TGF-β1 and MMP-2 and MMP-9 in tissue samples from keloids. Further study found that antisense TGF-β1 oligonucleotide treatment significantly decreased MMP-9 secretion, but had no effect on MMP-2 *in vitro* (Sadick et al., 2008). Activating transcription factor 3 (ATF3) is the ATF/CREB family and plays critical roles in modulating cellular behaviors by activating or repressing downstream genes (Wang et al., 2022). Wang et al. (2021) demonstrated the expression of ATF3 was upregulated in human keloid tissues. ATF3 has also been showed to suppress apoptosis and promote invasion of keloid fibroblast cells (Wang et al., 2021). In addition, upregulation of ATF3 significantly elevated the level of TGF-β1 and the phosphorylation of Smad2 and Smad3, while inhibition of ATF3 decreased TGF-β1 level and the phosphorylation of Smad2 and Smad3 in keloid fibroblast cells (Wang et al., 2021). Meanwhile, the mRNA and protein levels of MMP2 and MMP9 were elevated in ATF3-overexpressing cells, and ATF3 knockdown significantly downregulated MMP2 and MMP9 expression (Wang et al., 2021). Consistently, Jiang et al. (2016) also reported that growth differentiation factor-9 (GDF-9), a member of the TGF-β family, promoted the proliferation and migration of keloid fibroblasts by upregulating MMP-2 and MMP-9 expression, and enhancing Smad2 and Smad3 phosphorylation. Also, other studies reach similar conclusions (Bran et al., 2010; Lee et al., 2013; Zhao et al., 2019). Study showed that TGF-β1 controlled cell migration and invasion by regulating MMP-2 and MMP-9 activities (Muscella et al., 2020). These findings suggest that the TGF-β1/Smad signaling pathway may facilitate keloid growth by upregulating the expression of MMP-2 and MMP-9.

#### 3.1.2 Negative association between TGF-β1 and MMP-2/MMP-9

Conversely, the upregulation of MMP-2 and MMP-9 have also been reported to inhibit keloid formation. Silibinin, a natural polyphenolic flavonoid, has been reported to possess anti-inflammatory, antioxidant, antiapoptotic and anti-fibrotic properties (Tuli et al., 2021). Cho et al. (2013) demonstrated that silibinin induced the downregulation of type I collagen and inhibited the activation of Smad2/3. Meanwhile, silibinin significantly promoted the expression of MMP-2 (Cho et al., 2013). Therefore, silibinin may prevent fibrotic skin changes by downregulating type I collagen expression through the upregulation of MMP-2 and the inhibition of the Smad2/3 signaling pathway. Accumulating evidences have indicated that thalidomide, a-N-phthalimidoglutarimide, is important in fibrotic diseases, mainly due to its anti-fibrotic properties (Bajwah et al., 2013; Bose and Verstovsek, 2018). Liang et al. (2013) found that TGF-β1 could induce fibronectin expression in keloid fibroblasts and the effect was suppressed by pretreatment with thalidomide. In addition, pretreatment with thalidomide suppressed the TGF-β1-induced phosphorylation of Smad3 (Liang et al., 2013). Furthermore, thalidomide increased the activity of MMP-9, leading to fibronectin degradation (Liang et al., 2013). The findings are in consist with results obtained from results from Spiekman et al. (2014). These results indicated that thalidomide might inhibit keloids formation by upregulating MMP-9 expression through the inhibition of the TGF-β1/Smad3 signaling pathway.

In the early stage of skin wound healing, the expression of MMP-9 is important for the removal of ECM components from damaged tissues, which can help to create an environment conducive to cell migration and proliferation (Banerjee et al., 2024). TGF - β1 is also involved in the regulation of cell proliferation, differentiation, and migration during this time period. TGF - β1 can regulate the activity and expression of MMP-9 to a certain degree, so as to bring the remodeling of extracellular matrix into a dynamic balance, neither excessive degradation nor excessive deposition, and promote the normal repair of tissues (Li et al., 2022). TGF-β1 can regulate the activity and expression of MMP-9 to a certain extent, so that the remodeling of the ECM is in a state of dynamic equilibrium, neither over-degraded nor over-deposited, and the normal repair of tissues is promoted. MMP-2 is involved in the degradation of ECM, which is necessary for cell migration and tissue remodeling (D’Abadia et al., 2020). TGF-β1 can indirectly inhibit MMP-2 activity by upregulating the expression of TIMPs. A fine balance exists between TGF-β1 and MMP-2/MMP-9 to ensure that tissue repair occurs properly. Therefore, the relationship between them is not a simple positive or negative correlation, but a relationship of mutual cooperation and mutual constraint during keloid formation.

#### 3.1.3 Roles of TIMPs in keloid formation

TIMPs are the endogenous inhibitors of MMPs. Downregulation of TIMPs in keloid fibroblasts is found to elevate degradation of the excessive collagen bundles in keloid ECM. Aoki et al. found that the expression of MMP-2 was increased in keloids expressing small interfering RNA of TIMP-1 or TIMP-2, regulating ECM degradation and remodeling through the Collagen types I and III (Aoki et al., 2014). Fujiwara et al. (2005) revealed that keloid-derived fibroblasts exhibited an increased secretion of factors associated with collagen turnover and relied on matrix metalloproteinase (i.e., MMP-1 and MMP-2) for migration. Monocyte chemoattractant protein - 1 (MCP - 1), which is a C–C chemokine, has been demonstrated to prompt the recruitment of monocytes to the injured tissue and to play a crucial role in wound healing. Yeh et al. (2009) showed that IL-1β could induce a significant increase in MCP-1 and MMP-2 production in keloid-derived fibroblasts, which contributed to an imbalance in ECM formation and excess deposition of collagen in keloid. Imaizumi et al. (2009) reported that MMP-2 activity cooperated with TIMP-2 and MT1-MMP might contribute to the remodeling of collagen bundle areas and the invasion of fibroblasts into the surrounding normal regions via the promoted degradation of the ECM. Hepatocyte growth factor (HGF) functions to suppresses collagen synthesis. Lee et al. (2011) indicated that the enzymatic activities of MMP-2 was positively associated with HGF protein in the pathologic keloids, which was mediated by the regulation of type I and III collagen. Therefore, one of the main molecular mechanisms underlying the effects of MMP-2 and MMP-9 might attributed to their regulation on the ECM and collagens.

### 3.2 miR-188-5p inhibits keloids formation by suppressing MMP-2 and MMP-9 through inhibition of PI3K/AKT signaling pathway

Previous studies have shown that miRNAs may be involved in the development of keloids (Yu et al., 2015). Recent data have shown that miR-188-5p plays a crucial role in keloid formation. Vascular endothelial growth factor (VEGF), a specific provascular endothelial growth factor, is involved in keloids formation by modulating angiogenesis (Song et al., 2018). Zhou et al. (2022) reported that the inhibition of miR-188-5p promoted the proliferation, migration and cell cycle process, and inhibited the apoptosis of keloid fibroblasts. Furthermore, miR-188-5p inhibitor positively regulate VEGFA expression (Zhou et al., 2022). In addition, downregulation of VEGFA also abolished the promotive effect of miR-188-5p inhibitor (Zhou et al., 2022). Therefore, miR-188-5p may inhibit keloids formation by repressing the expression of VEGFA. It was reported miR-188-5p promoted tumor growth of pediatric acute promyelocytic leukemia by activating the PI3K/AKT signaling pathway (Wang et al., 2020). Yao et al. (2019) demonstrated that luteolin, a naturally occurring flavonoid, induced the apoptosis and inhibited the proliferation of human melanoma cells by decreasing the expressions of MMP-2 and MMP-9 via the PI3K/AKT pathway. A recent study from Zhu et al. (2019) indicated that miR-188-5p was significantly downregulated in keloid tissue compared with normal skin tissues. Upregulated expression of miR-188-5p inhibited keloids fibroblasts proliferation, migration, and invasion (Zhu et al., 2019). Furthermore, miR-188-5p mimics repressed the expression levels of MMP- 2, MMP-9, PI3K, and p-AKT in keloids fibroblasts (Zhu et al., 2019). In contrast, miR-188-5p inhibitor significantly increased the expression of MMP-2, MMP-9, PI3K, and p-AKT. Importantly, PI3K/AKT inhibitor reversed the promotive effect of miR-188-5p on MMP-2 and MMP-9 in keloids fibroblasts (Zhu et al., 2019). These findings together demonstrated that miR-188-5p inhibited keloids formation by suppressing PI3K/AKT/MMP-2/9 signaling pathway.

### 3.3 Platelets (PL) may promote keloid formation by upregulating MMP-9 expression through the regulation of p38 and ERK1/2 pathway

As is well known, growth factors play essential roles in the tissue neoformation and healing process (Miricescu et al., 2021). Growth factors are involved in many of the processes to tissue repair, including angiogenesis and cell proliferation, while they also influence l the synthesis and degradation of ECM proteins (Nagarkoti et al., 2023; Pot et al., 2023). Platelets contain different growth factors and cytokines, contributing to the formation of clot at sites of vascular injury by preventing blood loss (Schmidt et al., 2019). In the past several decades, the research on the physiological characteristics of platelets gradually deepened in tissue injury, which made it possible to treat keloids with platelets. A recent meta-analysis showed that platelet-rich plasma has a 23% response rate in the management of scars, and it were 22% and 23% in patients with laser or micro-needling, respectively (Ebrahimi et al., 2022). This suggests that platelet-rich plasma seems to be a safe and effective treatment for keloids. Tsai et al. (2023) reported that type A platelet-derived growth factor (PDGF-AA), an important growth factors in regulating cell growth and function, inhibits Leydig cell growth, migration, and invasion by activating ERK. In addition, PDGF has also been shown to facilitate the invasion and metastasis of cholangiocarcinoma cells by upregulating the expression of MMP-2/MMP-9 and inducing epithelial-mesenchymal transition (EMT) through activating the p38/MAPK signaling pathway (Pan et al., 2020). Scopelliti et al. (2020) demonstrated that platelet lysate (PL) promoted wound healing by increasing fibroblast production of ECM components and keratinocyte migration. However, whether PL is involved in the development of keloids remains unknown. Ranzato et al. (2011) showed that PL upregulated the expression of MMP-9 rather than MMP-2 in human keratinocyte cell line. Furthermore, both inhibitor of ERK1⁄2 pathway and inhibitor of p38 significantly inhibited MMP-9 activity induced by PL (Ranzato et al., 2011). As is well known, collagen type I is a major component of ECM and skin connective tissue, while collagen type III is secreted in the granulation tissue that is formed during wound healing (Li et al., 2023). PL has been reported to increase the production of collagen type III, but has no effect on the production of collagen type I (Ranzato et al., 2011). Taken together, PL may promote keratinocyte epithelialization and enhancing fibroblast matrix deposition by upregulating MMP-9 expression through p38 and ERK1/2 pathway, leading to keloid formation. Platelets initiate a cascade of events that lead to fibroblast activation and ECM production, while MMP - 2 and MMP - 9 play a role in the abnormal ECM remodeling and cell-related processes that are characteristic of keloid formation. The interplay between these factors is complex. At present, however, the available relevant studies were limited, which needs further validation in future.

### 3.4 LncRNA-ZNF252P-AS1/miR-15b-5p/BTF3 promotes keloid progression by up-regulating MMP2 and MMP9 through inhibiting JAK2/STAT3 signaling pathway

Keloids is highly heterogeneous and its cells display Warburg metabolism (Sun, 2022). Warburg metabolism was firstly found in neoplastic cells by Dr. Otto H (Li et al., 2020). Warburg and this discovery led to the awarding of the Nobel Prize (Li et al., 2020). Recently, JAK/STAT signaling pathways has been reported to be an inducer of Warburg metabolism. Chen et al. (2023) reported that tofacitinib decreased the volume and dermis thickness of the keloid by inhibiting fibroblast proliferation and collagen I synthesis through the suppression of STAT3. In addition, IL-6 (interleukin-6) and sIL-6r (soluble IL-6 receptor) are involved in joint cartilage destruction by stimulating the production of MMPs via JAK/STAT signaling pathway in chondrocytes (Aida et al., 2012). Zhou et al. (2020) found that JAK/STAT signaling pathway inhibitor inhibited the invasion and progression of keloid fibroblasts by downregulating the expression of MMP-2 and upregulating the expression of TIMP-2. Mounting studies have shown that microRNAs play an important role in the mechanism of keloid formation. Kuai and Jian (2022) demonstrated that miR-23b-3p was upregulated in keloid fibroblasts. Further study found that inhibition of miR-23b-3p significantly inhibited keloids by facilitating A20 expression (Kuai and Jian, 2022). The basic transcription factor 3 (BTF3) has been reported to be closely associated with cell proliferation and apoptosis. Wu et al. (2020) showed that the BTF3 promoted the migratory and invasive abilities of cervical cancer cells via interaction with MMP-2 and MMP-9. Recently, the expression of lncRNA-ZNF252P-AS1, pJAK2, p-STAT3, BTF3 MMP-2 and MMP-9 were found to be upregulated, whereas miR-15b-5p expression is downregulated in keloid tissue and keloid fibroblasts (Guo et al., 2022). Furthermore, miR-15b-5p overexpression significantly downregulated proliferation and migration ability of KFs, while this phenomenon was reversed by BTF3 overexpression (Guo et al., 2022). In addition, miR-15b-5p overexpression downregulated MMP-2, MMP-9 and collagen I protein levels, while the overexpression of BTF3 upregulated these proteins levels (Guo et al., 2022). Luciferase reporting experiments confirmed that BFT3 was targeted by miR-15b-5p and negatively modulated in keloid fibroblasts (Guo et al., 2022). Moreover, BTF3 knockdown has been reported to inhibit the JAK/STAT3 signaling pathway (Guo et al., 2022). It was reported that lncRNA-ZNF252P-AS1 overexpression significantly downregulated miR-15b-5p level and upregulated BTF3 level (Guo et al., 2022). Furthermore, lncRNA-ZNF252P-AS1 overexpression significantly increased the proliferation and migration ability of keloid fibroblasts and upregulated MMP-2 and MMP-9 levels (Guo et al., 2022). Importantly, silencing lncRNA-ZNF252P-AS1 inhibited keloid progression and decreased p-JAK2 and p-STAT3 expression (Guo et al., 2022). These studies suggested that lncRNA-ZNF252P-AS1/miR-15b-5p/BTF3 might promote keloid progression by up-regulating MMP2, MMP9 and collagen I protein levels through inhibiting JAK2/STAT3 signaling pathway. Therefore, inactivation of lncRNA-ZNF252P-AS1 may be a potential therapeutic target for keloid.

**FIGURE 2:**
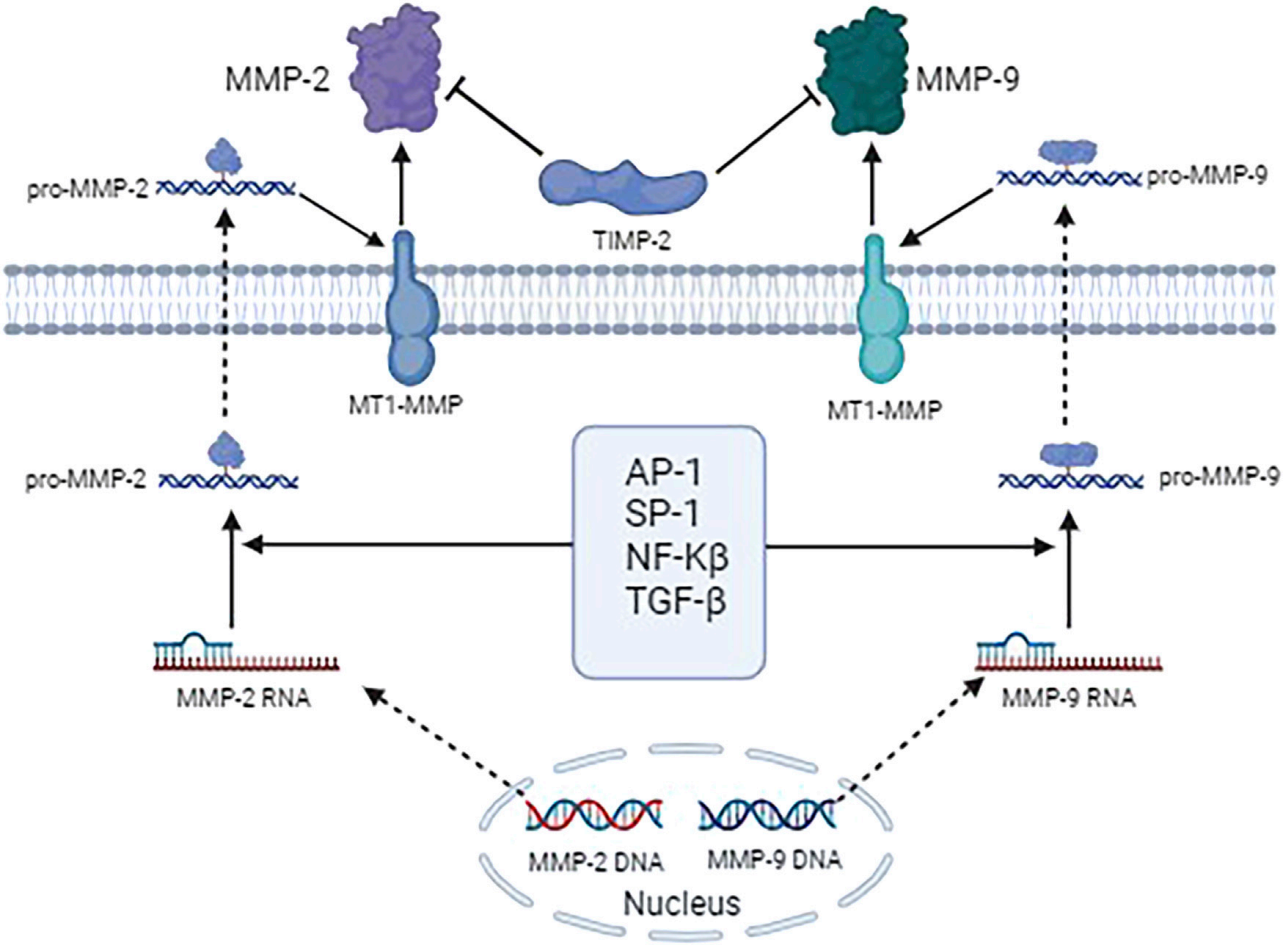
Molecular mechanisms of MMP-2 and MMP-9 and their associated genes and signal cascades in the pathological process of keloids.

Figure 2 shows the underlying molecular mechanisms of MMP-2 and MMP-9 and their associated genes and signal cascades in the pathological process of keloids.

### 3.5 Roles of MMP-2 and MMP-9 in some treatment modalities on keloids

Since both MMP-2 and MMP-9 have been found to be involved in the pathogenesis of keloids development, several potential drugs or substances that targeted the two genes are found to be effective on the treatments or preventions of keloids. Intralesional steroid injection (i.e., triamcinolone) is a widely used treatment for keloids. Besides, 5-fluorouracil (5-FU) has also been found to be one of the promising drugs for treating keloids. Huang et al. (2013) reported that a combination of triamcinolone and 5-FU could improve the scar regression and declined the recurrence of keloids by modulating keloid fibroblasts through the regulation of MMP-2 expression. Starfish hatching enzyme was reported to have diverse functions, including hydrolyze type I collagen. Li et al. (2014) demonstrated that the starfish hatching enzyme treatment could improve the scar and keloid by decreasing the proliferation of fibroblasts. Mechanistically, the starfish hatching enzyme exerted the anti-keloid effects by regulating the fibroblast-populated collagen gel conditions via the interaction of MMP-2 and MMP-9 and the inflammatory genes. Cryotherapy is also one of the promising therapeutic methods to treat keloid scars. Based on the observations of that CD163^+^ M2 macrophages and MMP-9 were dramatically elevated in cryotherapy-treated tissue, Lee et al. (2020) concluded that cryotherapy improved keloids by recruiting tissue re-modeling M2 macrophages with accompanying MMP-9. Dispel-Scar Ointment (DSO), a common-used in the traditional Chinese medicine, has been found to effectively treat keloids. Li et al. (2024) explored the molecular mechanisms underlying the influence of DSO on keloid by performing a network pharmacology, molecular docking analysis, and experiment validations. They found that MMP2-flavoxanthin, MMP9-luteolin, and MMP-9-kaempferol bound best to DSO, which might be associated with the reduction of TGF-β1, pSMAD2, and CoL1a1 expression. Tranilast, an anti-allergic agent, has been found to inhibit keloid and hypertrophic scar formation. Shimizu et al. (2006) implied that tranilast could suppress the formation of keloid scarring by inhibiting the expression of MMPs (i.e., MMP-7, MMP-8, and MMP-9) and TIMP (i.e., TIMP-1) in neutrophils. Clinical data showed that botulinum toxin type A (BTXA) could inhibit the development of hypertrophic scarring, while the potential mechanisms were unclear. Hao et al. (2018) found that BTXA promoted the healing of scars by suppressing the proliferation of keloid fibroblasts as well as regulating the expression of TGF-β1 and MMP-2. These studies demonstrated that the therapeutic effect of existing therapies for keloids might be partially attributed to the regulation of both MMP-2 and MMP-9. All the drugs and substances mentioned above were marketing approval. The researchers found that these drugs can alleviate keloids formation by elaborately regulating MMP-2 and MMP-9 expressions. However, the inhibitors of MMP-2 and MMP-9 for treating keloids are not yet available in human trials. Nevertheless, the present study revealed that developing more efficient drug delivery systems on MMP-2 and MMP-9 may be one of the successful treatments for managing keloids. Liposomes or polymeric nanoparticles can be designed to encapsulate anti-MMP2/9 drugs and deliver them directly to the fibroblasts or ECM in the keloid tissue. On the other hand, by analyzing the expression patterns of MMP-2, MMP-9 in a patient’s keloid tissue, it may be possible to predict the response to different pharmacological interventions and tailor the treatment accordingly.

## 4 Clinical research

In addition to the above experimental research, the roles of MMP-2 and MMP-9 have also been explored in clinical tissue specimens of keloids. A previous pilot study demonstrated that the medians levels of both MMP-2 and MMP-9 were increased in the hypertrophic scar and keloid groups as compared to the donor skin (Tanriverdi-Akhisaroglu et al., 2009). Paltatzidou et al. (2017) assessed the expression of MMP-9 in the lesional skin biopsies taken from patients who received 5-fluorouracil treatment with skin keloids. They found that MMP - 9 was strongly expressed in the multinuclear giant cells of keloid biopsies, while it was significantly decreased after adding cryotherapy (*P* < 0.05). The above results revealed that both MMP-2 and MMP-9 was validation by the clinical settings, which made it possible to achieve clinical transformation by targeting the two genes. Up to date, only two eligible studies belonging to clinical researches implied the roles of MMP-2/MMP-9 on keloid formation. Therefore, more future clinical studies are still warranted to better evaluate the association between MMP-2/MMP-9 and keloid formation.

## 5 Limitations

It is important to acknowledge certain limitations in the current body of literature, including variations in study methodologies and potential gaps in our understanding of the intricate signaling networks involved. Based on the above findings, MMP-9 expression may have contrasting effects on keloid formation. According to the experimental and clinical data, MMP-9 expression levels are usually elevated in keloid tissue. High level of MMP-9 is found to degrade collagen, elastin, and other components of the extracellular matrix and promotes the migration and proliferation of fibroblasts, which leads to the continuous expansion of keloid tissue. The expression levels of some inflammatory cytokines and growth factors are also often elevated in keloid tissues, and these factors may stimulate fibroblasts to secrete MMP-9, further exacerbating keloid fibrosis. Therefore, downregulating MMP-9 expression may significantly inhibit keloid development. For example, Zhou et al. (2022) found that miR-188-5p inhibited keloids formation by suppressing MMP-9 expression. However, a previous study conducted by Liang et al. (2013) demonstrated that thalidomide might inhibit keloids formation by upregulating MMP-9 expression. The inconsistent results from different studies might be attributed to different stages and progress of the disease, or the complexity of regulatory networks. Keloids formation is usually a dynamic process and different stages may involve different pathophysiologic mechanisms. In addition, MMP-9 may be regulated by multiple transcription factors, and different transcription factors may have different activities at different stages of keloids formation, causing the MMP-9 expression to exhibit opposite effects in this disease. As a result, more studies are still warranted to confirm the exact role of MMP-9 in the development and progression of keloid formation.

## 6 Conclusion

This comprehensive review extensively illustrates intricate roles of MMP-2 and MMP-9 in the regulation of keloids. The reviewed studies demonstrate elevated expression levels of MMP-2 and MMP-9 in keloid tissue compared to normal skin, suggesting their pivotal role in driving the excessive collagen deposition and altered tissue architecture observed in keloids. Furthermore, the interplay between MMP-2, MMP-9, and their regulators, such as TGF-β1/Smad, PI3K/AKT and LncRNA-ZNF252P-AS1/miR-15b-5p/BTF3 signaling pathways, highlights the intricate balance governing ECM homeostasis. Dysregulation of this balance not only underscores the significance of MMP-2 and MMP-9 but also opens new avenues for exploring targeted therapies for keloids. In summary, this review consolidates our current understanding of MMP-2 and MMP-9 in keloid pathogenesis, shedding light on their intricate involvement in the dysregulated keloids processes. The potential for targeted therapeutic interventions offers promising avenues for advancing keloid management strategies.
